# Complete genome sequence of the *Microbacterium* sp. strain BDGP8

**DOI:** 10.1128/MRA.00384-23

**Published:** 2023-08-21

**Authors:** Kenneth H. Wan, Soo Park, Benjamin W. Booth, Ella C. Brydon, Joanne Eichenberger, Jamie L. Inman, Jian-Hua Mao, Antoine M. Snijders, Susan E. Celniker

**Affiliations:** 1 Biological Systems and Engineering Division, Lawrence Berkeley National Laboratory, Berkeley, California, USA; Wellesley College, Wellesley, Massachusetts, USA

**Keywords:** genomes, non-human microbiome

## Abstract

*Microbacterium* sp. BDGP8 is a species of facultative anaerobic gram-positive bacterium of the family Microbacteriaceae. The complete genome consists of a single circular chromosome of 3,293,567 bp with a G + C content of 69.84% and two plasmids of 49,365 bp and 32,884 bp.

## ANNOUNCEMENT

The strain was isolated from a C57BL/6J mouse housed at Lawrence Berkeley National Laboratory under specific pathogen-free laboratory conditions. Two related organisms are a non-motile *Microbacterium paludicola* strain US15T, first isolated from soil in Korea (1) and *Microbacterium* sp*. SGAir 0570* isolated from air in Singapore (2). Our sequence is similar by average nucleotide identity (3) to the two sequenced *Microbacterium* species, *M. paludicola* strain CC3 (91.4%) (4) and *Microbacterium* sp. *SGAir 0570* (91.3%). Both are more similar to each other 99.1% than to our new isolate, *Microbacterium* sp. BDGP8.

A swab (sterile and saline soaked) of the left ear pinna of a 100-day-old, female mouse (C57BL/6J, The Jackson Laboratory, Sacramento, CA, USA) was directly plated onto a BHI (brain heart infusion) agar plate and grown at 30°C for 24 h. A single colony was inoculated into 5 mL of BHI and incubated at 30°C for 18 h. A bacterial pellet was resuspended and amplified by 16S PCR (V1-V4) and Sanger sequenced to identify species type. Genomic DNA (gDNA) from a *Microbacterium* sp. BDGP8 colony-purified overnight culture was isolated using Qiagen kits (Cat. Nos. 19060, lysis buffers and 10262, DNA purification tips) and sequenced on Oxford Nanopore MinION and Illumina MiSeq platforms. Nanopore libraries were constructed using the rapid barcoding kit (SQK-RBK-004) with barcode RB02; multiplex sequenced on an FLO-MIN106D (R9.4.1) flow cell using MinKNOW v.4.5.4; and basecalled and demultiplexed with Guppy (5.1.13 + b292f4d, Fast model, 450bps). Illumina libraries were constructed by shearing gDNA via sonication (Diagenode Bioruptor), followed by library preparation with the NEBNext Ultra DNA (NEB E7370) and Multiplex Oligo (NEB 7600) kits using i709 and i505 primers with eight rounds of amplification. Illumina libraries were multiplexed and paired-end sequenced on a v2 500-cycle nano flow cell (Cat. No. MS-103-1003), followed by a v3 600-cycle flow cell (Cat. No. MS-102-3003) for deeper coverage. Default parameters were used for all software tools.

Nanopore sequencing yielded 238,738 reads with N50 read length of 5,785 bp, totaling 714.9 Mbp. Illumina sequencing yielded 1,441,953 reads, totaling 416.9 Mbp. *De novo* assembly was performed using Unicycler v0.4.9b (5) with 94.67% Nanopore and 99.7% Illumina reads mapping, with an estimated coverage of 194× and 230×, respectively. Sequence quality was assessed using Minimap2 (6).

We analyzed and annotated the assembly using RAST v2.0 (7) and NCBI PGAP v4.11 (8). The NCBI annotation predicts 3,277 genes in total; 3,221 protein-coding genes, 56 RNA genes including 2 rRNA operons; 3 ncRNAs and 47 tRNA genes. We discovered two incomplete partial prophages (18.7 kb and 15.2 kb) that contain 23 genes for capsid and tail assembly suggesting the original bacteriophage was a tailed phage belonging to the order *Caudovirales*.

The *M. paludicola* strain US15T is described as non-motile. Using the RAST annotation, we identified in *Microbacterium* sp. BDGP8 a flagellar gene operon spanning 27,747 bp for which a Kyoto Encyclopedia of Genes and Genomes (KEGG) analysis (9) identifies 26 genes (flgB-E, flgK-L flgN, flhA-B, fliD-K, fliM, fliN-S, and motA-B) of 54 that encode and regulate flagellar assembly and function. We found four genes not shown in the KEGG pathway flaA (filament protein), flbD (transcriptional regulatory protein), fliW (assembly factor), and an RNA polymerase sigma factor for the flagellar operon. These findings suggest that the BDGP8 strain may demonstrate independent flagellar-propelled locomotion.

## Data Availability

*Microbacterium* sp. BDGP8 and the two plasmids are deposited in GenBank under accession no. CP120712 to CP120714. The SRA accession numbers are SRR23890202 to SRR23890204. The sequences of the two strains used for comparison are *Microbacterium paludicola* strain CC3 (CP018134.1) and *Microbacterium* sp. SGAir 0570 (CP027929.1). The sequence of *Microbacterium paludicola* US15T 16s rRNA (AJ853909).
